# Justice in priority-setting for research on health and climate change

**DOI:** 10.2471/BLT.25.294480

**Published:** 2026-03-30

**Authors:** Soumyadeep Bhaumik

**Affiliations:** a Meta-research and Evidence Synthesis Unit, Health Systems Science, The George Institute for Global Health, University of New South Wales, Health Translation Hub, 8 / 55 Botany St, Randwick, NSW 2031, Australia.

Traditionally, the academic community has prioritized the academic value of research over its social impact and relevance. About a decade ago, a study estimated that up to 85% of biomedical research was avoidably wasted. This study highlighted that a key factor contributing to research waste was the limited utility and relevance of research for interest-holders, particularly for health workers, patients, caregivers and communities.^1^ To address research waste, funders are increasingly conducting research priority-setting exercises, a formal systematic process through which interest-holders determine what research should be prioritized.^2^ Research priority-setting is important for epistemic reasons (that is, relating to knowledge) but also for moral and pragmatic purposes.^3^ Morally, people have the right to be involved in making decisions related to their health. From a pragmatic standpoint, involving interest-holders facilitates resource mobilization and reinforces accountability. 

Many methods for conducting research priority-setting exercises exist, some more systematic than others and each with their own strengths and limitations.^2^ The importance of ethical principles in research priority-setting is highlighted in the World Health Organization (WHO) document *WHO* g*uidance on the ethics of health research priority-setting*.^3^ However, conceptualizing research priority-setting only as a technical tool for identifying priority topics prevents it from realizing its full potential to advance justice in knowledge production systems. Advancing justice is particularly important for climate and health research because various scholars have shown that climate change, and actions to address climate change, embody multiple dimensions of injustice. These dimensions include but are not limited to the disproportionate harms to those least responsible, limited participation of marginalized groups in decision-making to protect their environment and rising inequity. Therefore, the knowledge developed to address climate change and its health and social impacts should aim to mitigate, if not redress, these injustices. 

Here, I examine and describe the dimensions of justice in setting priorities for research on health and climate change using the weaving approach,^4^^,^^5^ which is common in Eastern and Indigenous knowledge traditions. Rather than proposing new conceptual frameworks, this approach integrates established concepts from diverse domains to clarify their intersections. Weaving also enables the pursuit of shared meaning on the topic of enquiry among all those involved or interested. This epistemological stance offers a distinct contribution, by fostering dialogue and debate across disciplines and knowledge systems. Drawing on Western constructs of justice and global health ethics, this article weaves concepts together in a nonprescriptive manner. This approach invites readers to apply their own worldviews to the individual dimensions of justice while exploring their relationalities. 

## Dimensions of justice

Climate change is one of the greatest threats to humankind. The convergence of climate change with health is not only a scientific challenge; it has profound ethical and moral implications. The health impacts of climate change disproportionally affect different nations, communities and individuals. However, most climate and health research is conducted in and by high-income countries. Even within countries, different geographies, populations and individuals have different risks because of the complex interplay of vulnerability, exposure and hazards. Communities that are least responsible for climate change are often the most exposed to its consequences. This imbalance underscores the need for justice to serve as a guiding principle in setting research agendas on climate and health. Using justice as a guiding principle in research priority-setting in health and climate change allows researchers to use knowledge that addresses these injustices, aligning with the well-established importance of justice in climate change.^6^

The principle of justice is deeply rooted across cultures, with its meaning inherently understood but its application requires articulating its many dimensions. Five dimensions of justice are relevant to research priority-setting on climate and health. First, procedural justice, which examines whether the process for setting research priorities is fair and appropriate. Procedural justice requires fair process, inclusivity of interest-holders, transparency and accountability. Methodological guidance of research priority-setting primarily focuses on aspects of procedural justice. 

Second, epistemic justice, which relates to whose knowledge and knowledge systems are recognized and valued in the research priority-setting exercise, as well as the research being undertaken; it has two elements: testimonial justice and hermeneutical justice.^5^ Testimonial injustice refers to the credibility deficit assigned to certain groups, which may result in tokenistic (that is, superficial rather than meaningful) involvement or exclusion from leadership roles or authorship in research priority-setting exercises. Hermeneutical injustice arises when a lack of shared interpretive resources (that is, social understanding) prevents people from nondominant communities from articulating their unique experiences, preferences and values. In research priority-setting exercises, this type of injustice may be reflected in the exclusion of traditional medicine; failure to recognize Eastern or Indigenous understanding of the interconnected nature of the health of human, animals and nature; or the use of substantive criteria for setting priorities that are difficult for non-scientists to interpret. 

Third, intergenerational justice, which explores who bears the burden of health impacts of climate change that spans across generations. Considering intergenerational justice would require research priority-setting exercises to consider rights, health and well-being of future generations by focusing on research that aims to mitigate climate change and its health impacts.^6^^,^^7^

Fourth, distributive justice, which refers to the fair allocation of resources and the benefits of research to those who are marginalized. To do so, research priority-setting exercises should consider equity as a key factor and determine in which locations the research is being conducted. Previous work on transnational global health research has outlined inequities in research funding, authorship and global knowledge production.^5^^,^^7^^–^^10^

Fifth, restorative justice, which examines how the conduct and practice of research address historical harms. Nations and communities who are facing most climate-related health impacts without being historical contributors of climate change should not only be provided resources for research to understand and address it but also lead its conduct. Reparative funding to build research infrastructure and capacity instead of project-based funding for climate change and health research in an autonomous manner (for example, *Swaraj* or self-governance) is a potential pathway for restorative justice. Rooted in anti-colonial philosophy, *Swaraj*^11^ calls for communities, particularly those disproportionately affected, to lead the processes for defining and addressing their own research needs. The polluter-pays principle offers a clear framework for assigning responsibility for mitigation, adaptation and loss and damage. However, in practice this principle is inconsistently applied, resulting in an incomplete account of who should bear the climate change burdens.^12^

If justice dimensions are considered in a research priority-setting exercise, the process as well as its output will be fair, ultimately transforming the knowledge system on climate change and health to a pro-justice one. A hypothetical example of what pro-justice research priority-setting entails is presented in Table 1. This reframing shifts the research from a purely technical process to one that examines equity, recognizes systemic barriers, and considers social determinants of health as well as promotes justice and fairness in the knowledge generation process. Doing so requires conducting formative work to deepen the understanding of what pro-justice research priority-setting entails, and developing frameworks and tools with procedural and substantive guidance through research with diverse interest-holders.

**Table 1:** Example of how a research topic on health and climate can be reframed in a research priority-setting exercise using a pro-justice framing

Without explicit justice framing	Pro-justice framing
**Research priority-setting output**	
Evaluate the effectiveness of cooling centres in reducing heat-related mortality and morbidity in urban areas	Evaluate cultural appropriateness, opportunity cost and effectiveness and equity implications of cooling centres in urban areas, with a focus on informal workers, migrants and women. Studies conducted in smaller urban cities, with principal investigator living in the same urban city, and give high priority to representatives from trade unions and women’s rights organizations as co-investigators
**Research priority-setting evaluation**	
Evaluation criteria: • impact on decision-making, allocation of funding and/or resources and research output • process evaluation of the research priority-setting to understand stakeholder satisfaction, reliability, usefulness and innovation potential	Additional justice-sensitive criteria to be included in the evaluation: • procedural (community participation) • epistemic (knowledge plurality) • distributive (benefits to those most affected) • intergenerational (long-term resilience and impact) • restorative (correcting historical injustices)

## Conclusion

Societal values regarding climate change and health are shaped by awareness of past and present injustices. In the current knowledge system, practices that encourage geographical and social separation between researchers and the communities they study reduce the social value of research and hinder interest-holders from meeting obligations to local communities.^3^^,^^8^^,^^10^^,^^13^ Therefore, research in climate and health cannot achieve its larger purpose without considering layered dimensions of injustice; doing so requires going beyond asking what research needs to be conducted. When justice is considered in research priority-setting exercises, it can become a transformative tool that challenges structures, amplifies marginalized voices and promotes equity,^14^ justice and accountability within the knowledge system.

The effort to reframe research priority-setting from a technical exercise to a justice-oriented social process is not isolated, it aligns with other pro-justice initiatives such as transitioning to shared health governance (shared ideals of global justice, sovereignty, resources and responsibility, and mutual collective accountability);^8^ promoting solidarity as a foundational value in academia;^15^ and structural reforms in the knowledge system.^13^

The pursuit of justice and knowledge have long been intertwined, reflecting diverse traditions and cultures. The weaving of justice in our global knowledge system on climate and health is and should remain a pursuit that requires collective resolve, and not just in research priority-setting.
